# The Application of Deep Learning Tools on Medical Reports to Optimize the Input of an Atrial-Fibrillation-Recurrence Predictive Model

**DOI:** 10.3390/jcm14072297

**Published:** 2025-03-27

**Authors:** Alain García-Olea, Ane G Domingo-Aldama, Marcos Merino, Koldo Gojenola, Josu Goikoetxea, Aitziber Atutxa, José Miguel Ormaetxe

**Affiliations:** 1 Biobizkaia Research Institute, Basurto University Hospital, 48013 Bilbao, Spain; 2 Hitz Group, Bilbao School of Engineering, University of the Basque Country, 48940 Bilbao, Spain

**Keywords:** atrial fibrillation, deep learning, natural language processing, Artificial Intelligence

## Abstract

**Background**: Artificial Intelligence (AI) techniques, particularly Deep Learning (DL) and Natural Language Processing (NLP), have seen exponential growth in the biomedical field. This study focuses on enhancing predictive models for atrial fibrillation (AF) recurrence by extracting valuable data from electronic health records (EHRs) and unstructured medical reports. Although existing models show promise, their reliability is hampered by inaccuracies in coded data, with significant false positives and false negatives impacting their performance. To address this, the authors propose an automated system using DL and NLP techniques to process medical reports, extracting key predictive variables, and identifying new AF cases. The main purpose is to improve dataset reliability so future predictive models can respond more accurately **Methods and Results**: The study analyzed over one million discharge reports, applying regular expressions and DL tools to extract variables and identify AF onset. The performance of DL models, particularly a feedforward neural network combined with tf-idf, demonstrated high accuracy (0.986) in predicting AF onset. The application of DL tools on unstructured text reduced the error rate in AF identification by 50%, achieving an error rate of less than 2%. **Conclusions**: This work underscores the potential of AI in optimizing dataset accuracy to develop predictive models and consequently improving the healthcare predictions, offering valuable insights for research groups utilizing secondary data for predictive analytics in this particular setting.

## 1. Introduction

**Figure 1:**
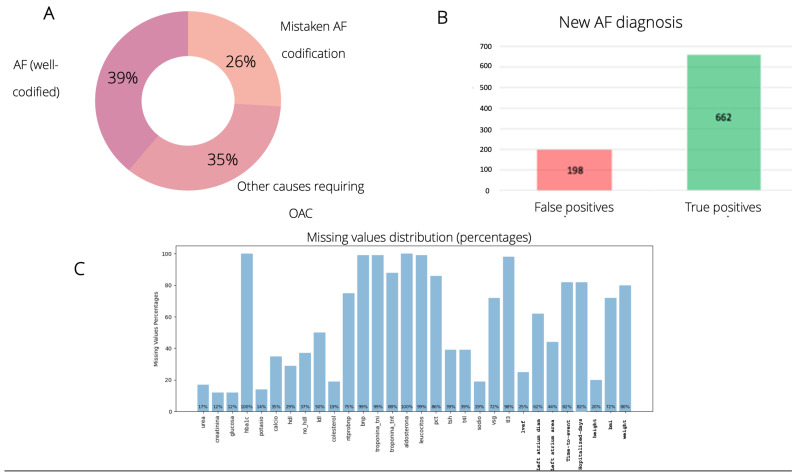
Main problems in extracting secondary data through hospital coding in the dataset audit. (**A**) False negatives (loss of introduction in the AF debut model) (**B**) False positives (previous diagnoses, incorrect coding ) (**C**) Percentages of missing values. AF, atrial fibrillation; OAC, oral anticoagulation.

Artificial Intelligence (AI) techniques have experienced exponential growth in the biomedical field in recent years. These tools are used in the development of predictive models based on secondary population data present in electronic health records (EHRs), given the advantage of sample representativeness compared to conventional trials and registries. The main limitation of these models is the reliability of the Big Data that feeds the algorithms. In previous work by this group, an explanatory predictive model based on machine learning (ML) was developed, predicting the recurrence or persistence of atrial fibrillation (AF) over two years with an area under the curve of 0.71 in an internal validation [1]. In an internal dataset audit, twenty-three percent of the data obtained through EHR coding were false positives, which were removed through manual review, and a cross-check with patients who started anticoagulation within the same timeframe revealed a 26% deficit in coding and the consequent exclusion of AF episodes (false negatives). Additionally, for each instance, there were missing values among the predictive variables (between 12% and 100%), which led to lower predictive reliability (Figure 1). As a consequence, the use of secondary coded data was considered suboptimal for the development of AF recurrence predictive algorithms.

### Electronic Health Records Data Optimization Approaches

As hospital codification might present the aforementioned weaknesses, among others [2], several research groups have explored the utility of various tools applied to free-text medical data.

In this context, Abbas et al. [3] used regular expressions—character sequences used to define search patterns in texts—over clinical free-text to identify arrhythmia diagnoses. Regular expressions enhance the capability of detecting uncodified data, thereby increasing the sensitivity of data acquisition.

Significantly, in recent years, Language Models, AI models trained with large volumes of data and focused on NLP tasks, have experienced immense development. These models have already demonstrated their usefulness in processing medical reports [4], both pre-trained [5] and “de novo” trained [6] models, and their clinical and research opportunities are expanding.

Consequently, this background leads to the hypothesis that extracting unstructured (uncoded) data from medical reports could increase the sensitivity and specificity of instance and variable acquisition to feed our dataset. This, ultimately, could optimize the accuracy of the predictive model for AF recurrence prediction.

## 2. The Purpose of the Study

Two main objectives were established. First, to develop an automated system that, by applying applying natural language processing (NLP) and Deep Learning (DL) techniques, is capable of processing medical reports and extracting information from variables to be included in the predictive model. Second, to detect new cases of atrial fibrillation (AF) from medical reports using NLP techniques. A secondary objective was to compare the discrimination of a NLP model for AF detection by feeding the algorithm with highly specific or highly sensitive data.

## 3. Methods

The clinical data-extraction method was evaluated by an independent organization, receiving a favorable impact evaluation. The Basurto University Hospital Ethical Committee supported the study under the code 99.20CEICHUB and approved the informed consent exemption under the code 106.21CEICHUB. The project adhered to good clinical practice directives and complied with the 2016/627 European Regulation. Following approval by the center’s Ethical Committee, 1,210,736 discharge reports in unstructured format (free text in *.txt* format), generated from 2015 to 2020, were pseudonymized and extracted.

At the same time, through exhaustive search filters, instances coded as new cases of AF and the corresponding information regarding 114 potential predictive variables were extracted from the center’s coded system in the same period. These variables are listed the Supplementary Material File S1—Variables list.

Regular expressions (*regex*, a NLP technique that performs searches for specific terms) were then applied to discharge reports. This tool searched for the same variables analyzed in the coded system in particular sections of the reports. To do so, these reports were segmented by previously validated FLAIR models [2]. After the application of regex tools on discharge reports, both new cases of AF and potential recurrence predictive variables were elucidated and compared to encoded data.

A manual review of both EHR and reports in a sample of 150 instances coded by the system was performed as the gold standard method to check the reliability of the AI tools applied to reports.

Finally, a test was conducted to determine the optimal data source for AI data analysis. Deep Learning tools were applied to two groups of reports: one in which new cases of AF were identified by both regular expressions and the coding system, and another where these new cases were identified only by one of the methods.

All code used in this study was implemented in Python version 3.9 (Python Software Foundation, Beaverton, OR, USA). The vector generation process relies on the `re’ library, which is specialized in regular expressions. Feedforward models were trained using the `scikit-learn’ library, while text vectorization was performed using `NLTK’ and `Gensim’, or `scikit-learn”, depending on the selected technique—word embeddings or TF-IDF, respectively. Flair models were trained using the official `Flair’ Python library, whereas language models were trained with the `Transformers’ library. Training of language models was conducted on GPUs to optimize performance. All code is publicly available at https://github.com/anegda/PRAFAI-Debut (accessed on 12 January 2024).

### 3.1. Data Preprocessing

A Flair-based section identification model [7] was used to segment clinical reports into standardized sections: Header, Reason for Consultation, Personal History, Current Illness, General Exploration, Complementary Tests, Diagnosis, Treatment, and Evolution. This segmentation enabled the extraction of specific variables, ensuring proper differentiation between, for example, current and past test results or family medical history and the patient’s personal medical history.

**Figure 2:**
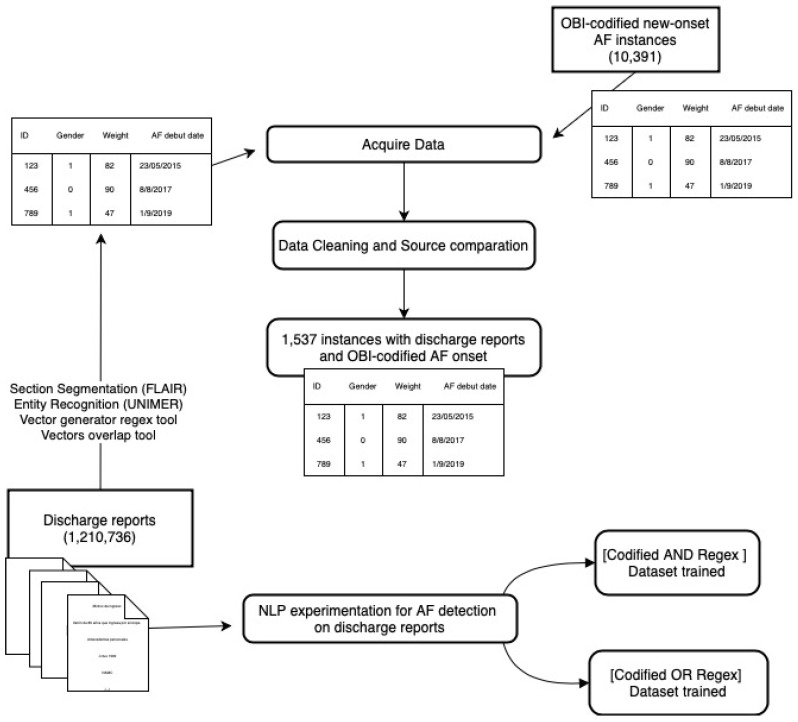
Methods Diagram.

A Medical Entity Recognizer (UNIMER) [8] was applied to detect medical entities (e.g., diseases, medications) and their relationships within the text, with a particular focus on its negation extraction module to prevent false positives in disease identification and test results. This tool combines Flair models for entity detection and negation recognition with a transformer-based relation detector (Figure 2).

### 3.2. Automatic Vector Generation Process

A custom script was developed to extract variables of interest from the preprocessed reports.

Tailored regex patterns were created for each variable based on synonyms and common expressions. A concise word root selection was made by cardiologists in the study to optimize the data input. These were applied to relevant report sections to detect mentions of variables.

Different extraction strategies were used based on variable type. Demographics were extracted from the Reason for Consultation and Current Illness sections. Medical history was searched for in the Personal History and Diagnosis sections (excluding AF). Lab-test numeric values were extracted from the Complementary Tests section. Medications recognized by UNIMER were mapped to ATC codes, as the OBI automatic system used them as well. Procedures/interventions were detected in relevant sections using regex.

UNIMER’s negation detection was leveraged to avoid false positives.

Special attention was given to detecting new AF diagnoses and avoiding prior AF history by checking mentions in different sections.

### 3.3. Vector Overlapping

**Figure 3:**
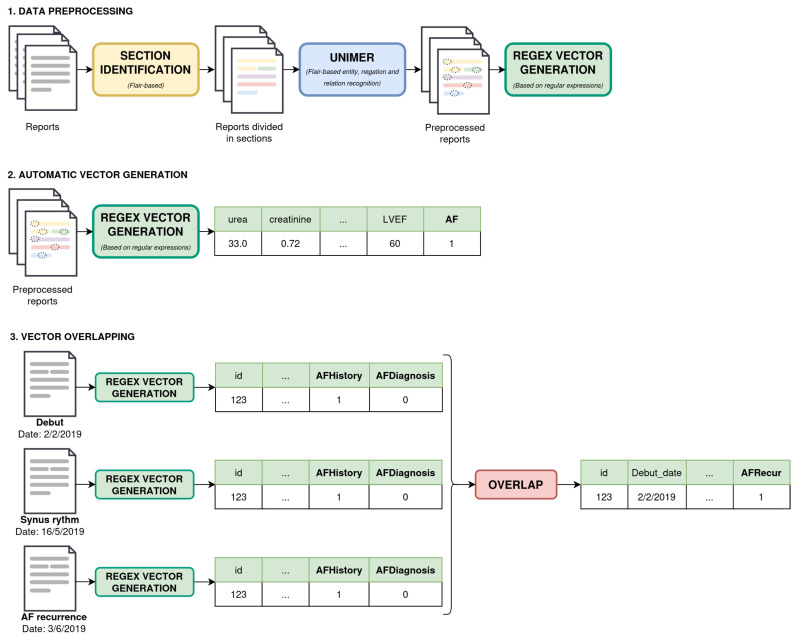
An exemplification of the three-step process for vector generation.

To generate more complete patient-specific vectors, information from multiple reports is required. Consequently, individual report vectors were generated for all available reports of a patient. Using the Overlap tool developed in this study, data from multiple reports were merged into a single vector that accurately represents the patient’s health status around the AF diagnosis date (Figure 3). This tool incorporates the following considerations:

- Pre-specified date ranges for different variable types (6 months prior for lab tests, following or previous 3 months for echo parameters) were used to optimize the clinical data reliability.
- In cases where numerous data were found for the same variable, reports closest to the debut date were prioritized.
- The AF diagnosis flags were concisely reviewed across reports to determine which reports contained the real new-onset diagnosis and which ones contained previous AF history and possible AF recurrence.

### 3.4. Vectors Evaluation

Two evaluation approaches were used: manual annotation and comparison to codified OBI vectors.

#### 3.4.1. Manual Annotation

A total of 26 reports (5 with AF information) were manually annotated. Custom accuracy, precision, recall, and F1-score metrics were defined to compare extracted and annotated variables. This led to a first comparison of how regex tools performed compared to expert clinician manual annotation of variables.

#### 3.4.2. Comparison to Codified OBI Vectors

Automatically generated vectors were compared to the coded OBI vectors to check whether these tools outperformed the coded data extraction. As not every coded data point was present in discharge reports, an overall comparison of missing data was performed.

### 3.5. Data Source Suitability for Data Analysys

To verify that regex tools conferred added value to the automatic AF detection in encoded data, a Deep Learning experiment was conducted.

Ten Natural Language Processing models were tested for automatic AF detection over those discharge reports identified by both codified data and regex tools (intersection) or those only detected by either method (union). The discrimination of Deep Learning algorithms for AF detection on discharge reports was compared in terms of accuracy, precision, recall, and F1-score between the two groups. Paired T-tests were used to compare the performance of NLP models trained with with highly specific data (intersection) and more sensitive data (union). In addition, the better performing NLP model was also compared with paired T-tests to the existing Flair model (with classic embeddings) for AF identification.

This methodology combines rule-based and machine learning NLP techniques to automate the extraction of structured medical data from unstructured clinical text. Section identification and entity recognition provide a foundation for more targeted information extraction using regular expressions. The vector overlapping approach aims to generate patient-level data comparable to manually coded OBI vectors.

## 4. Results

A total of 10,391 instances of patients with new-onset atrial fibrillation were obtained from the coded system between 2015 and 2020. In a sample of 150 manually reviewed patients from this global set, 117 instances of true new-onset atrial fibrillation were identified. Regular expressions correctly identified 88.23% of AF onsets in this subgroup, considering that not all patients with AF onset had a discharge report with the diagnosis.

The set of 10,391 instances coded as AF onset was then analyzed with this tool. Among them, 2778 reports were found around the dates of AF onset coding after duplicate instances were eliminated. Regular expressions were used over this report selection, which were close (30 days) to the coding date of the arrhythmia onset, along with a group of 11,000 random negative reports obtained from the universe of reports, identifying an intersection (match between coded and found by regular expressions) of 1537 AF onset reports and 5994 negative reports for AF onset.

Deep Learning models (multilayer neural networks with forward and backward propagation and language models based on LSTM—long-short term memory—and transformer architecture) were applied to this intersection group. The model consisting of a forward neural network combined with the tf-idf method (based on term frequency in the text) demonstrated the best AF onset prediction rate, with an accuracy, precision, recall, and F1-score of 0.986.

**Figure 4:**
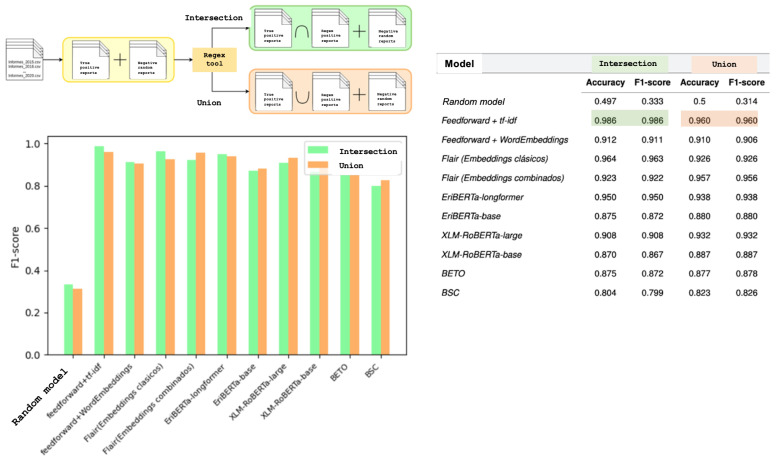
Model-training comparison after training with most specific (intersection) or sensitive (union) subgroups with overperformance of the first one.

When compared to the model generated by these tools on the union set (AF onsets identified either by regular expressions in the report or only by hospital coding), the error was almost 4% (50% higher) (Figure 4). A paired T-test between tf-idf in the Union set and tf-idf in the Intersection set showed a *p* = 0.0039, which is statistically significant. Notably, a paired T-test between feedforward + tf-idf (in the Intersection set) and Flair (with classic embeddings) showed a *p* = 0.0034, also significant and favorable for the specifically trained tool.

**Figure 5:**
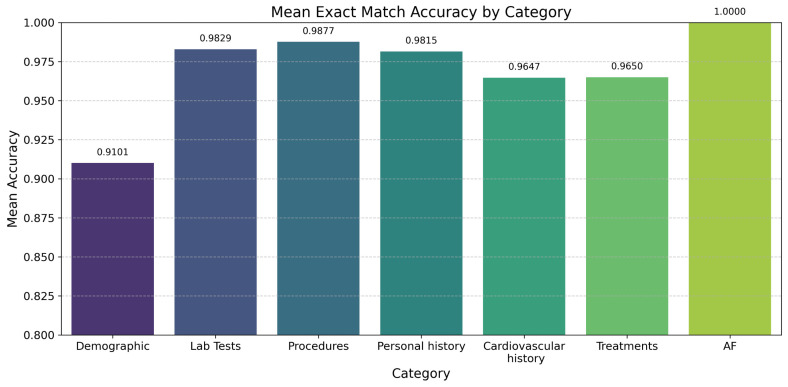
Mean accuracy for regex tool according to variables of interest.

Regarding the extraction of the 114 variables for each instance to be included in the future in the predictive model of AF recurrence, the regular expressions showed an average accuracy of 0.97 and a precision of 0.93 in a review of 30 manually reviewed reports, varying for each type of variable (Figure 5).

The application of regular expressions on the discharge reports from the electronic medical record resulted in a significant reduction (58.1%) in the number of missing values in the coded analytical variables (*p* < 0.005). Furthermore, given the identification of non-coded values such as echocardiographic parameters, a substantial improvement in the integrity and completeness of the data was observed.

## 5. Conclusions

Supervised machine learning algorithms are very useful for designing predictive models with optimal representativeness of the population on which healthcare activity is conducted, but their reliability depends on the accuracy of the data-feeding algorithms. This work demonstrates how regular expressions applied to medical reports can minimize missing data that limit the predictive power of machine learning algorithms. Indeed, it shows how applying Deep Learning tools on free medical text reduces the error in identifying patients with AF onset by 50% when trained with a highly specific dataset outperforming a more sensitive one. Finally, the application of Deep Learning algorithms on medical reports predicts with an error of less than 2% that the patient has a true onset of atrial fibrillation.

In the recent literature, EHRs were the most commonly used data for NLP model training [9], while data imbalance, domain adaptation, and transfer learning are assessed as some of the main challenges [10]. It is probable that the high representativeness of data reports, due to the inclusion of overall population reports, as well as the algorithm-specific training on the report structure, supported the NLP performance in our study.

### 5.1. The Impact of the Study

Data reliability is a cornerstone for training AI models in medical prediction, particularly when these models are intended for clinical practice. The quality, accuracy, and representativeness of clinical data directly affect the performance, safety, and trustworthiness of AI-driven prediction tools in healthcare.

Reliable data ensures that AI models can make accurate and consistent predictions. In clinical Cardiology, this translates to precise diagnoses, effective treatment recommendations, and early disease detection or recurrence prediction. For instance, if an AI model is trained on incomplete or inaccurate datasets, it may produce flawed outputs, leading to potential incorrect AF recurrence predictions and clinical decisions.

According to the sensitivity and specificity increase in this study, the generalization of AI models must be emphasized. Clinical data must represent diverse patient populations to prevent bias and ensure the AI model’s applicability across different demographics and healthcare settings. Poorly curated or biased datasets can lead to algorithms that perform well in one context but fail in others, such as when patient populations differ by age, comorbidities, gender, or other features.

This study also shows that reliable data enables faster processing and analysis during studies, and automation through AI may minimize manual errors in data management in this setting.

Consequently, we consider this work of particular interest to any research group using secondary data for the development and validation of predictive models, not limited to atrial fibrillation or AI studies.

### 5.2. Future Directions

Once the reliability of this AF onset dataset has been achieved with significant insight, AF recurrence prediction models must be adjusted. For AF recurrence prediction, NLP-based AF identification in consecutive reports should be explored, although gold-standard manual EHR review remains crucial for AF recurrence prediction, as discharge reports may not include every detail in the patient’s personal history. Indeed, newer AF ablation techniques (pulsed-field ablation) might lead to different blanking periods [11], in which recurrence should be cautiously managed.

A significantly higher performance is expected compared to previous models, as the reduction in missing values and higher data reliability should enhance its efficiency. In contrast, lower prediction accuracy might be achieved; and given the demonstrated enrichment of the dataset, this would show that (1) AF recurrence prediction is more difficult than expected and (2) imprecise data could lead to over-optimistic conclusions.

## Data Availability

Data are unavailable due to privacy and ethical restrictions. All code is publicly available at https://github.com/anegda/PRAFAI-Debut (accessed on 12 January 2024).

## Supplementary Materials

The following supporting information can be downloaded at: https://www.mdpi.com/article/10.3390/jcm14072297/s1, Table S1: List of variables.
