# Supplementary material for: The Application of Deep Learning Tools on Medical Reports to Optimize the Input of an Atrial-Fibrillation-Recurrence Predictive Model
Source: J Clin Med. 2025 Mar 27;14(7):2297. doi: 10.3390/jcm14072297 (PMC11989490; doi:10.3390/jcm14072297)
Supplement: Supplementary file 1 [file jcm-14-02297-s001.zip › jcm-3520267-supplementary.pdf]

Supplementary material Table S1 – Variables list

Identification
Date of AF debut
Gender
Age
Pensioner (0/1)
Resided (0/1)
Size (m)
Body mass index
Weight (kg)

<u>*Analytic variables*</u>
Serum urea (mg/dL)
Serum creatinin (mg/dL)
Serum albumin (g/dL)
Glucose (mg/dL)
Glicated hemoglobin -HbA1c- (mM, percentage)
Serum potassium (mEq/L)
Serum sodium (mEq/L)
Serum calcium (mg/dL)
HDL-colesterol (mg/dL)
Non-HDL cholesterol (mg/dL)
LDL-cholesterol (mg/dL)
Total cholesterol (mg/dL)
NT-proBNP (pg/mL)
BNP (pg/mL)
Ultra-sensitive T troponin (ng/mL)
Ultra-sensitive I troponin (ng/mL)
D-dimer (ng/mL)
Fibrinolgen (mg/dL)
Aldosterone (ng/dL)
Leukocytes (cells/mcL)
Procalcitonin (µg/L)
C reactive protein (mg/L)
TSH (mU/L)
Free T4 (ng/dL)
Free T3 (ng/dL)
Globular sedimentation speed (mm)

<u>*Procedures*</u>
Echocardiogram (0/1)
Left ventricular ejection fraction (%)
Left atrium diameter (mm)
Left atrium area (mm^2)
Constrast echocardiogram (0/1)
Electrocardiogram (01)
Cardioversion (0/1)
Pulmonary vein ablation (0/1)
Cardiac endovascular device (0/1)

<u>*Admissions*</u>
Number of admissions (n)
Cardiolovascular cause mediated admissions (n)
Number of days of the admission (n)
Number of days from admission to AF recurrence (n)

<u>*Personal history (0/1)*</u>
Depression

Alcohol consumption
Drug dependence
Anxiety
Dementia
Kidney failure
Menopause
Osteoporosis
Type 1 diabetes
Type 2 diabetes
Dyslipidemia
Hypercholesterolemia
Atrial flutter
Heart failure
Smoker
Obstructive apnea
Hyperthyroidism
Metabolic syndrome
Arterial hypertension
Ischemic heart disease
Strocke
Cardiomyopathy
Other arrhythmias
Psychogenic rhythm
Bigeminism
Branch locks
Atrioventricular blockade
Bradycardia
Premature extrasystolia
Sinus arrhythmia
Rheumatic mitral valve disease
Other valve diseases
Congenital valve diseases
Peripheral arteriopathy
Chronic Obstructive Pulmonary Disease

Treatment (ATC codes, 0/1)
b01a
n02ba01
a02bc
c03
g03a
a10
n06a
n05a
n05b
c01
c01b
c02
c04
c07
c08
c09
c10
Polimedication (0/1)

Type of Atrial fibrillation (paroxysmal/persistent/permanent)
Atrial fibrillation diagnosis in the text (0/1)
Previous diagnosed atrial fibrillation (0/1)
